# Repeat Colonoscopy within 6 Months after Initial Outpatient Colonoscopy in Ontario: A Population-Based Cross-Sectional Study

**DOI:** 10.1155/2017/5917057

**Published:** 2017-09-07

**Authors:** Lawrence Paszat, Rinku Sutradhar, Nancy N. Baxter, Jill Tinmouth, Linda Rabeneck

**Affiliations:** ^1^Institute for Clinical Evaluative Sciences, University of Toronto, G106 2075 Bayview Avenue, Toronto, ON, Canada M4N 3M5; ^2^Department of Surgery and Li Ka Shing Knowledge Institute, St. Michael's Hospital, University of Toronto, 30 Bond Street, Toronto, ON, Canada M5B 1W8; ^3^Sunnybrook Health Sciences Centre, University of Toronto, 2075 Bayview Avenue, Toronto, ON, Canada M4N 3M5; ^4^Prevention and Cancer Control, Cancer Care Ontario, University of Toronto, 620 University Avenue, Toronto, ON, Canada M5G 2L7

## Abstract

**Background:**

The goal of this study is to examine utilization of early repeat colonoscopy ≤ 6 months after an index procedure.

**Methods:**

We identified persons having repeat colonoscopy ≤ 6 months following outpatient colonoscopy without prior colonoscopy ≤ 5 years or prior diagnosis of colorectal cancer (CRC). We modeled repeat colonoscopy using a generalized estimating equation with an exchangeable correlation structure to account for clustering of patients by endoscopist.

**Results:**

The population included 334,663 persons, 7,892 (2.36%) of whom had an early repeat colonoscopy within 6 months. Overall, endoscopist prior year colonoscopy volume was inversely related to repeat ≤ 6 months. Repeat colonoscopy ≤ 6 months varied by the clinical setting of the index colonoscopy (adjusted OR = 1.41 (95% CI 1.29–1.55)) at nonhospital facilities compared to teaching or community hospitals. Among those who had polypectomy or biopsy, the adjusted OR for early repeat ≤ 6 months was elevated among those whose index colonoscopy was at a nonhospital facility (OR 1.44, 95% CI 1.30–1.60), compared to those at a teaching hospital or community hospital.

**Conclusions:**

Repeat colonoscopy ≤ 6 months after an index procedure is associated with the clinical setting of the index colonoscopy.

## 1. Introduction

Colonoscopy is an important procedure in the diagnosis, treatment, and surveillance of diseases of the large intestine, including the prevention of, and screening for, colorectal cancer (CRC). Colonoscopy in Ontario, Canada, is funded by the single, universal, government payer for health care services. The total number of colonoscopies in Ontario per year is constrained by the number of qualified endoscopists, the allocation of resources to colonoscopy within hospitals, and the level of funding for costs other than physician remuneration at nonhospital facilities. The provincial colorectal screening program, ColonCancerCheck (CCC) [1], recommends screening by colonoscopy for individuals at elevated risk for CRC (11% of population [2]) and by guaiac fecal occult blood testing (gFOBT) for individuals at average risk (89% of population). CCC coexists alongside opportunistic colonoscopic screening of persons not having elevated risk for CRC, consuming a substantial proportion of the provincial colonoscopy capacity. CCC will replace gFOBT by the fecal immunochemical test, whose higher sensitivity will place increasing demand upon the constrained colonoscopy resource.

We have recently shown the coexistence of inappropriate under- and overutilization of colonoscopy in Ontario, for example, suboptimal rates of colonoscopic follow-up of positive gFOBT among participants in the provincial colorectal screening program [3] and high rates of repeat colonoscopy less than five years after complete negative colonoscopy [4]. Overutilization of colonoscopy has been reported in other jurisdictions [5, 6], although there are very few publications including any evaluation of the use colonoscopy ≤ 6 months following an index procedure. The goal of this study is to examine utilization of early repeat colonoscopy ≤ 6 months after an index procedure, in an attempt to identify potential opportunities to reduce its frequency.

## 2. Materials and Methods

This study was approved by the Research Ethics Board of Sunnybrook Health Sciences Centre and conducted at the Institute for Clinical Evaluative Sciences (ICES).

### 2.1. Study Design

We conducted a population-based cross-sectional study of patients who underwent colonoscopy in Ontario between January 1, 2013, and June 30, 2014.

### 2.2. Data Sources

The Ontario Health Insurance Plan Database (OHIP) contains records of billing claims submitted by physicians, which include a descriptor of the service provided, the date of the service, and the unique identifier of the physician who provided the service. The Registered Persons Database (RPDB) contains time periods of OHIP eligibility and demographic information including postal and residence codes which are linkable to ecologic information about small-area median household income for all OHIP beneficiaries. The Corporate Providers Database is maintained by the Institute for Clinical Evaluative Sciences and contains demographic and practice-related information about Ontario physicians and surgeons. The Ontario Cancer Registry (OCR) contains the diagnosis code and date of diagnosis of invasive cancers diagnosed in Ontario between January 1, 1964, and December 31, 2014. The Ontario files of the Canadian Institute for Health Information (CIHI) (Discharge Abstract Database, Same Day Surgery Database, and National Ambulatory Care Recording System Database) contain dates of hospital visits and admissions, hospital identifiers, diagnosis codes using the International Classification of Diseases version 10, and procedure codes using the Canadian Classification of Interventions.

### 2.3. Identification of Study Cohort

We identified all persons who had at least one colonoscopy in the OHIP physician billing claims database between January 1, 2013, and June 30, 2014.

### 2.4. Covariates

#### 2.4.1. Patient Covariates

Age, sex, and postal or residence code were obtained from the RPDB. Postal or residence codes were linked to ecologic data from Statistics Canada on urban quintile of median household income/rural residence. The count of Johns Hopkins Aggregated Diagnosis Groups (the ADG score) during the 12 months prior to the date of colonoscopy was tabulated from diagnosis codes contained in the CIHI and OHIP databases for each patient [7–9]; a higher score reflects a higher burden of comorbidity [8].

#### 2.4.2. Colonoscopy Covariates

*Clinical Setting*. The clinical setting, in which the index colonoscopy was performed, was identified using the hospital identifier in the CIHI databases, to determine if the setting was a teaching or a community hospital. If there was only an OHIP billing claim and no CIHI record of the colonoscopy, clinical setting was labeled as a nonhospital facility [10].

*Completeness*. Each colonoscopy was categorized as complete or incomplete based on the presence or absence of OHIP fee codes E747 (indicating endoscopy to caecum) or E705 (indicating endoscopy to terminal ileum).

*Open Access*. Colonoscopy was labeled “open access” if there were no billing claims in OHIP for consultations or visits with the physician who billed OHIP for the colonoscopy during the 12 preceding months [11].

*Complexity of Procedures during Index Colonoscopy*. We categorized all colonoscopies into one of four hierarchical groups of procedural complexity by identifying procedures performed during the colonoscopies using OHIP fee codes that were billed on the same patient and service date as the colonoscopy. The highest category “removal of large polyp or obstructing lesion” comprised colonoscopies accompanied by any of the following four fee codes: E687 (excision of obstructing lesion by laser), Z764 (excision of obstructing lesion < 2 cm), Z765 (excision of obstructing lesion ≥ 2 cm), or E685 (excision of sessile polyp > 3 cm diameter). Accordingly, this category includes those for whom piecemeal resections were performed. The second highest category, “standard polypectomy,” comprised colonoscopies that did not include “removal of large polyp or obstructing lesion” but were accompanied by Z571 (excision of polyp) or Z570 (fulguration of polyp). The third highest category comprised colonoscopies that included the fee code E717 (biopsy) but no fee code for any type of polypectomy. The lowest category comprised colonoscopies that did not include any of the above procedures.

#### 2.4.3. Endoscopist Covariates

We identified the unique physician identifier from each colonoscopy billing claim and then extracted the sex and specialty (gastroenterology, general surgery, general internal medicine, and others) from the Corporate Providers Database. For each unique physician identifier, we extracted from the OHIP database all billing claims for each colonoscopy performed during the prior year (without exclusions) and the completeness of each colonoscopy and computed the prior year colonoscopy volume and prior year cecal intubation rate. We also extracted all billing claims for “removal of large polyp or obstructing lesion” (E685, E687, Z764, and Z765) and all billing claims for standard polypectomy (Z570, Z571), performed during the prior year, and computed the prior year volume for “removal of large polyp or obstructing lesion” and the prior year standard polypectomy volume for each endoscopist.

### 2.5. Analysis

The primary aim was to examine factors associated with the odds of receiving a repeat colonoscopy ≤ 6 months following an index colonoscopy. Univariate and multivariate logistic regression models were implemented, and a generalized estimating equations approach under an exchangeable correlation structure was used to account for potential clustering among patients by endoscopist [12]. As associations between characteristics and the odds of receiving a very early repeat colonoscopy may vary based on location of index colonoscopy, all analyses were stratified by the 3 categories of this variable (teaching hospital, community hospital, and nonhospital). Normality of continuous covariates was explored by assessing quantile-quantile plots against the normal distribution. A natural logarithm transformation was imposed on physician prior year volume of “removal of large polyp or obstructing lesion,” and a square root transformation was imposed on physician prior year colonoscopy volume and physician prior year standard polypectomy volume. Analyses of the secondary objectives were also conducted using the same methodological approach. Analyses were conducted with SAS version 9.3 (SAS Institute, Inc., Cary, NC). All statistical tests were 2 sided, and* P* values less than 0.05 were considered statistically significant.

## 3. Results

Among the 641,098 persons who underwent a colonoscopy in Ontario between January 1, 2013, and June 30, 2014, 334,663 were eligible for the study. We excluded persons (1) < 50 years old or >79 years old (*n* = 162,195), (2) those with a colonoscopy during the prior 5 years (*n* = 158,649), (3) those with colonoscopy during an inpatient admission (*n* = 17,265), (4) those with intraoperative colonoscopy (*n* = 168), (5) those with colonoscopy at an unknown facility (*n* = 3), (6) those with a prior diagnosis of CRC (*n* = 20,798) or a diagnosis of CRC on the date of the colonoscopy or ≤ 6 months thereafter (*n* = 7,471), (7) those without postal code or residence code in the RPDB for linkage to socioeconomic variables (*n* = 1,905), and (8) those with < 6 months follow-up in the RPDB after colonoscopy due to death or emigration (*n* = 5,391). Some persons had more than one exclusion factor.

Descriptive analyses were stratified by clinical setting of the index colonoscopy because of differences in patient, procedure, and endoscopist factors among teaching hospitals, community hospitals, and nonhospital facilities (Table 1). Patients in the nonhospital facilities tended to be younger and have a lower ADG score. The percent with complete colonoscopy, open access colonoscopy, and no polypectomy or biopsy significantly varied among the clinical settings (Table 1). The number of endoscopists performing the index colonoscopies during the 18-month eligibility period was 1,025, the majority of whom performed colonoscopies in at least two different clinical settings (25.8% at teaching hospitals, 69.1% at community hospitals, and 67.1% at nonhospital facilities). Endoscopist prior year colonoscopy and polypectomy volumes were highest among those whose practice included the nonhospital setting.

Within 6 months after the index colonoscopy, 7,892 persons underwent an early repeat colonoscopy (2.4%): 2.7% of those with index colonoscopy at a teaching hospital, 2.1% at a community hospital, and 2.6% at a nonhospital facility.

Patients having an early repeat colonoscopy were more likely to be male and older, with a higher ADG score (reflecting a higher burden of comorbidity) compared to the overall study population, and to have had an incomplete colonoscopy, a removal of a large polyp or obstructing lesion, or standard polypectomy at the index procedure. The percent of all those with incomplete colonoscopy at baseline who had a repeat ≤ 6 months was 16.3% (1,096/6,709). The percent of all those with a “removal of a large polyp or obstructing lesion” who had a repeat ≤ 6 months was 30.5% (647/2,121), the percent of all those with standard polypectomy was 4.3% (2,441/57,347), the percent of all those with a biopsy was 3.2% (1,174/36,528), and the percent of all those without any polypectomy or biopsy was 1.5% (3,630/238,667). Patient and procedure factors among those with a repeat varied by clinical setting of the index colonoscopy (Table 2).

Patients whose index colonoscopy was at a nonhospital facility and who had a repeat ≤ 6 months were more likely to have had no polypectomy or biopsy at the index (50.8% compared to 43.3% at a teaching hospital and 42.9% at a community hospital) (Table 2), were more likely to have had no polypectomy or biopsy at either index or repeat colonoscopy, 37.6%, (1,146/3,047) compared to 25.7%, (245/955) at a teaching hospital and 29.1%, (1,131/3,890) at a community hospital, and were more likely to have the repeat in a different clinical setting (36.0% compared to 13.4% of those whose index colonoscopy was performed at a teaching hospital and 13.5% at a community hospital). The repeat procedure was performed by a different endoscopist for 30.1% of those with index colonoscopy at a nonhospital facility, compared to 22.3% of those at a community hospital and 33.6% of those at a teaching hospital.

The endoscopist prior year volumes and prior year cecal intubation rate were not normally distributed (data not shown) so transformations were applied to achieve a normal distribution for the adjusted analyses. Endoscopist prior cecal intubation rate and completeness of the index colonoscopy were collinear; therefore cecal intubation rate was excluded from the adjusted analyses.

Overall, and stratified by clinical setting of index colonoscopy, the highest adjusted odds ratios for repeat ≤6 months were for removal of a large polyp or obstructing lesion performed at the index colonoscopy and incompleteness of the index colonoscopy (Table 3).

In the overall analysis adjusting for patient, colonoscopy, and endoscopist factors, patients who had an index colonoscopy at a nonhospital facility were more likely to have a repeat ≤ 6 months (adjusted OR = 1.41 (95% CI 1.29–1.55)), even though those patients had a lower risk of colorectal pathology (younger) and had a higher percent with complete colonoscopy and lower percent with any procedure performed at either the index or repeat colonoscopy. In the stratified analysis among those with index colonoscopy at nonhospital facilities, endoscopist factors were not associated with the likelihood of repeat ≤ 6 months. Open access colonoscopy was not associated with repeat ≤ 6 months in any clinical setting.

Similarly, among all patients with any polypectomy or biopsy, those who had an index colonoscopy at a nonhospital facility were more likely to have a repeat ≤ 6 months (adjusted OR = 1.44) (95% CI 1.30, 1.60) (Table 4).

Among all patients with a repeat ≤ 6 months, those with an index colonoscopy at a nonhospital facility were less likely to have the repeat performed by the same endoscopist who performed the index colonoscopy (adjusted OR = 0.71) (95% CI 0.60, 0.83) (Table 5).

## 4. Discussion

Early repeat colonoscopy ≤ 6 months following an index outpatient colonoscopy in Ontario is associated with appropriate indications such as incompleteness, or removal of a large polyp or obstructing lesion, at the index procedure. Early repeat procedures are also more likely if the index procedure has been performed in a nonhospital setting, despite the younger lower risk population in that setting and lower rate of polypectomies and biopsies, and is not associated with endoscopist factors in this setting. In our prior work, the odds ratio of a repeat colonoscopy < 5 years after a negative complete colonoscopy was also elevated if the baseline colonoscopy had been performed in a nonhospital setting [4]. Early repeat colonoscopy after an index procedure at a nonhospital setting is more likely to occur in a different clinical setting and to be performed by a different endoscopist than repeat colonoscopy after index procedures performed in hospital settings.

In Ontario, the percent of colonoscopies performed in the nonhospital setting is increasing and has risen from 18.9% between the years 2000 and 2007 in our previous publication [4] to 35.1% in this study during 2013-2014. The efficiency and effectiveness of colonoscopy in this setting will be increasingly important as the guaiac fecal occult blood test is replaced by the fecal immunochemical test, and as the average age of the Ontario population steadily increases.

The US Multisociety Task Force (USMSTF) guidelines for colonoscopy are based on risk stratification for colorectal neoplasia determined by a high quality index colonoscopy, with clearing of the colon [13]. More than one colonoscopy may be required to achieve complete clearing with high confidence. In addition, if an adenoma is removed in piecemeal resection, the USMSTF recommends a short interval for repeat colonoscopy. In the 2006 guidelines the recommendation was 2–6 months [13] and in the 2012 updated guidelines the recommendation was < 1 year [14]. If the bowel prep at the index colonoscopy is poor, the USMSTF recommends that the procedure should be repeated within 1 year. Finally, some patients may have had a FOBT+ after the index colonoscopy and before a scheduled surveillance colonoscopy. In these cases there is no clear recommendation and decision making is individualized.

Very few publications have reported the frequency of early repeat colonoscopy ≤ 6 months following an index procedure, and those that have provided this information were primarily focused on repeat colonoscopy within 3 or 4 years following a screening colonoscopy [15, 16]. Stock et al. [15] reported 1.0% of patients had a repeat ≤ 6 months following a screening colonoscopy; Pyenson et al. [16] do not cite a percent but from figure 1 in this publication fewer than 1.0% had an early repeat procedure. We cannot estimate the proportion of repeat procedures that were inappropriate or avoidable, or the proportion of patients who would have benefitted from a repeat procedure but did not receive one, due to the limitations of available data. We do not know the clinical indication for colonoscopy. We have no information about the adequacy of bowel preparation. We have no information about the histopathological diagnosis of polypectomy or biopsy specimens, other than the absence of invasive carcinoma, and cannot distinguish adenoma from hyperplastic lesions. We have no information about the number of lesions or the completeness of resection. Optimization of the efficiency and effectiveness of colonoscopy across clinical settings will require higher quality data on key elements, including indication, bowel preparation, and histopathology. Nevertheless, the increased likelihood of early repeat colonoscopy at nonhospital facilities, adjusting for known patient and procedural factors, suggests that efforts to reduce the frequency of early repeat colonoscopy at those facilities would be reasonable.

Focusing on understanding early repeat colonoscopy is important for two reasons. First, to the extent that these procedures are not aligned with clinical guidelines, they reflect overuse. It is also possible that there are some patients who should have an early repeat procedure, for whom none is provided. Second, colonoscopy is associated with risk for harms, including bleeding perforation and even death [17]. It may be possible to reduce the frequency of early repeat colonoscopy by adopting a suite of measures aimed at improving the quality of colonoscopy, including adherence to guidelines for surveillance colonoscopy [18], improving bowel preparation by patient education [19], and split dose-bowel preparation [20], as well as measures to improve the completeness, the adenoma detection rate, and the rate of complete of polyp resection at the baseline colonoscopy [20].

## 5. Conclusions

After adjusting for patient factors, early repeat colonoscopy ≤ 6 months after an index procedure is associated with the clinical setting of the index colonoscopy, which could be targeted for intervention to reduce the frequency of avoidable early repeat procedures.

## Figures and Tables

**Table 1:** Study population and characteristics of index colonoscopy.

	Clinical setting of index colonoscopy				Entire population
	Teaching hospital	Community hospital	Nonhospital facility		
Number of patients	35,988 (10.8%)	181,039 (54.1%)	117,636 (35.1%)		334,663

*Patient factors*				*P* value among strata	
Age					
50–54 years	8,722 (24.2%)	45,437 (25.1%)	39,953 (34.0%)	<0.0001	94,112 (28.1%)
55–59 years	7,423 (20.6%)	37,220 (20.6%)	26,255 (22.3%)		70,898 (21.2%)
60–64 years	7,039 (19.6%)	33,708 (18.6%)	21,102 (17.9%)		61,849 (18.5%)
65–69 years	6,003 (16.7%)	30,313 (16.7%)	16,406 (14.0%)		52,722 (15.8%)
70–74 years	4,212 (11.7%)	21,050 (11.6%)	9,520 (8.1%)		34,782 (10.4%)
75–79 years	2,589 (7.2%)	13,311 (7.4%)	4,400 (3.7%)		20,300 (6.1%)

Female	19,984 (55.5%)	95,409 (52.7%)	59,675 (50.7%)	<0.0001	175,068 (52.3%)
Male	16,004 (44.5%)	85,630 (47.3%)	57,961 (49.3%)		159,595 (47.7%)

Residence					
Income quintile 1	5,368 (14.9%)	20,683 (11.42%)	15,596 (13.3%)	<0.0001	41,647 (12.4%)
Income quintile 2	5,986 (16.6%)	25,722 (14.2%)	19,832 (16.9%)		51,400 (15.4%)
Income quintile 3	6,000 (16.7%)	29,277 (16.2%)	22,191 (18.9%)		57,468 (17.2%)
Income quintile 4	6,838 (19.0%)	34,405 (19.0%)	25,812 (21.9%)		67,055 (20.0%)
Income quintile 5	9,121 (25.3%)	35,006 (19.3%)	29,915 (25.4%)		74,042 (22.1%)
Rural	2,675 (7.4%)	35,946 (19.9%)	4,290 (3.7%)		42,911 (12.8%)

ADG score					
Mean (SD)	4.91 (3.01)	4.88 (2.82)	4.24 (2.63)	<0.0001	4.72 (2.79)
Median (IQR)	4 (3, 7)	4 (3, 7)	4 (2, 6)		4 (3–6)

*Colonoscopy factors*					
Complete	34,844 (96.8%)	177,338 (98.0%)	115,772 (98.4%)	<0.0001	327,954 (98.0%)
Incomplete	1,144 (3.2%)	3,701 (2.0%)	1,864 (1.6%)		6,709 (2.0%)

Open access	21,584 (60.0%)	58,834 (32.5%)	85,148 (72.4%)	<0.0001	165,566 (49.5%)
Not open access	14,404 (40.0%)	122,205 (67.5%)	32,488 (27.6%)		169,097 (50.5%)

Most complex procedure at index colonoscopy					
“Removal of large polyp or obstructing lesion”	228 (0.6%)	1,284 (0.7%)	609 (0.5%)	<0.0001	2,121 (0.6%)
Standard polypectomy	6,060 (16.8%)	32,412 (17.9%)	18,875 (16.1%)		57,347 (17.1%)
Biopsy	6,368 (17.7%)	21,195 (11.7%)	8,965 (7.6%)		36,528 (10.9%)
None of the above or none	23,332 (64.8%)	126,148 (69.7%)	89,187 (75.8%)		238,667 (71.3%)

*Endoscopist factors* ^*∗*^					
Female	68 (24.3%)	145 (19.5%)	123 (17.3%)	0.05	215 (20.9%)
Male	212 (75.7%)	597 (80.5%)	579 (81.8%)		810 (78.6%)

Endoscopist specialty					
Gastroenterology	110 (39.3%)	159 (21.4%)	196 (27.5%)	<0.0001	250 (24.4%)
General surgery	117 (41.2%)	420 (56.6%)	351 (49.3%)		512 (49.9%)
Internal medicine	43 (15.4%)	76 (10.2%)	108 (15.2%)		130 (12.7%)
Other	10 (3.6%)	87 (11.7%)	57 (8.0%)		133 (13.0%)

*Endoscopist prior year practice factors*					
Prior year colonoscopy volume	Mean: 406.6	533.6	Mean: 554.9	<0.0001	Mean 468.8
	(SD: 439.0)	(SD 419.7)	(SD: 433.2)		(SD 407.2)
	Median 305	Median 455	Median 492		Median 384
	(IQR 94–559)	(IQR 214–755)	(IQR 224–755)		(IQR 150–661)

Prior year volume “removal of large polyp or obstructing lesion”	Mean: 3.8	Mean: 3.9	Mean: 4.5	<0.0001	Mean 3.5
	(SD: 11.8)	(SD: 11.1)	(SD: 12.3)		(SD 10.7)
	Median 0	Median: 0	Median: 1		Median 0
	(IQR 0–3)	IQR (0–4)	(IQR 0–4)		(IQR 0–3)

Prior year standard polypectomy volume	Mean: 132.5	Mean: 188.8	Mean: 195.5	<0.0001	Mean 163.4
	(SD: 179)	(SD: 185.6)	(SD: 199.9)		(SD 182.7)
	Median 85	Median: 136	Median: 142		Median 110
	(IQR 25–168)	(IQR 62–260)	(IQR 61–275)		(IQR 38–232)

Prior year cecal intubation rate	Mean 0.92	Mean 0.95	Mean 0.95	<0.0001	Mean 0.94
	(SD 0.09)	(SD 0.07)	(SD 0.09)		(SD 0.09)
	Median 0.94	Median 0.96	Median 0.96		Median 0.96
	(IQR 0.91–0.97)	(IQR 0.94–0.98)	(IQR 0.93–0.98)		(IQR 0.93–0.98)

^**∗**^(The majority of endoscopists performed colonoscopy at two or more settings.)

**Table 2:** Very early repeat colonoscopy: patient characteristics and description of index colonoscopy (7,892).

Clinical setting of index colonoscopy	Teaching hospital	Community hospital	Nonhospital facility	
	955/35,988 (2.7%)	3,890/181,039 (2.1%)	3,047/117,636 (2.6%)	7,892/334,663

*Patient characteristics*				*P* value among strata
Age				
50–54 years	175 (18.3%)	707 (18.2%)	777 (25.5%)	<0.0001
55–59 years	166 (17.4%)	715 (18.4%)	625 (20.5%)	
60–64 years	180 (18.9%)	741 (19.1%)	629 (20.6%)	
65–69 years	181 (19.0%)	776 (20.0%)	528 (17.3%)	
70–74 years	154 (16.1%)	555 (14.3%)	322 (10.6%)	
75–79 years	99 (10.4%)	396 (10.2%)	166 (5.5%)	

Female	445 (46.6%)	1651 (42.4%)	1290 (42.3%)	0.05
Male	510 (53.4%)	2239 (57.6%)	1757 (57.7%)	

Residence				
Income quintile 1	174 (18.2%)	529 (13.6%)	451 (14.8%)	<0.0001
Income quintile 2	156 (16.3%)	598 (15.4%)	531 (17.4%)	
Income quintile 3	163 (17.1%)	630 (16.2%)	587 (19.3%)	
Income quintile 4	179 (18.7%)	680 (17.5%)	657 (21.6%)	
Income quintile 5	199 (20.8%)	638 (16.4%)	692 (22.7%)	
Rural	84 (8.8%)	815 (21.0%)	129 (4.2%)	

ADG score				
Mean (SD)	5.93 (3.49)	5.44 (3.09)	4.83 (2.84)	<0.0001
Median (IQR)	5 (3–8)	5 (3–7)	4 (3–6)	

*Colonoscopy description*				
Complete	770 (80.6%)	3409 (87.6%)	2617 (85.9%)	<0.0001
Incomplete	185 (19.4%)	481 (12.4%)	430 (14.1%)	

Open access	564 (59.1%)	1193 (30.7%)	2172 (71.3%)	<0.0001
Not open access	391 (40.9%)	2697 (69.3%)	875 (28.7%)	

Most complex procedure at index colonoscopy				
Removal of large polyp or obstructing lesion	59 (6.2%)	407 (10.5%)	181 (5.9%)	<0.0001
Standard polypectomy	318 (33.3%)	1247 (32.1%)	876 (28.8%)	
Biopsy	165 (17.3%)	567 (14.6%)	442 (14.5%)	
None of the above or none	413 (43.3%)	1669 (42.9%)	1548 (50.8%)	

**Table 3:** Adjusted odds ratios for very early repeat colonoscopy^#^.

		Clinical setting of index colonoscopy		
	Overall	Teaching hospital	Community hospital	Nonhospital facility
	OR (95% CI)	OR (95% CI)	OR (95% CI)	OR (95% CI)
*Patient factors*				
Patient age (per one year increase)	1.01 (1.01, 1.02)	1.01 (1.00, 1.02)	1.01 (1.01, 1.02)	1.02 (1.01, 1.02)
Patient sex				
Female	0.70 (0.66, 0.73)	0.69 (0.60, 0.80)	0.67 (0.62, 0.71)	0.73 (0.68, 0.79)
Male	Reference	Reference	Reference	Reference
Patient ADG score^*∗*^	1.05 (1.04, 1.06)	1.08 (1.06, 1.10)	1.05 (1.04, 1.07)	1.02 (1.02, 1.04)

*Index colonoscopy factors*				
Setting				
Teaching hospital	1.03 (0.91, 1.18)	Not applicable	Not applicable	Not applicable
Community hospital	Reference			
Nonhospital facility	1.41 (1.29, 1.55)			
Complete	Reference	Reference	Reference	Reference
Incomplete	10.92 (9.89, 12.06)	9.49 (7.80, 11.54)	9.50 (8.28, 10.93)	14.60 (12.19, 17.49)

*Most complex procedure at index colonoscopy*				
Removal of large polyp or obstructing lesion	30.39 (25.62, 36.04)	19.94 (12.99, 30.61)	40.07 (33.15, 48.43)	23.54 (17.26, 32.11)
Standard polypectomy	2.82 (2.63, 3.03)	2.96 (2.45, 3.57)	2.82 (2.56, 3.11)	2.86 (2.52, 3.25)
Biopsy	2.40 (2.17, 2.66)	1.43 (1.12, 1.83)	2.30 (1.99, 2.66)	3.34 (2.82, 3.95)
None of the above	Reference	Reference	Reference	Reference

*Index endoscopist factors (prior year volumes)*				
Colonoscopy volume^*∗*1^	0.98 (0.97, 0.99)	0.95 (0.93, 0.97)	0.94 (0.91, 0.98)	1.00 (0.98, 1.02)
Standard polypectomy volume^*∗*1^	1.03 (1.01, 1.04)	1.06 (1.02, 1.10)	1.05 (1.03, 1.07)	1.01 (0.98, 1.04)
Large or obstructing polypectomy volume^*∗*2^	0.98 (0.96, 1.01)	1.00 (0.95, 1.06)	0.94 (0.91, 0.98)	1.01 (0.97, 1.05)

^*∗*^Per one unit increase; ^1^square root transformation; ^2^log transformation; ^#^(adjusted for patient socioeconomic status, colonoscopy open versus closed access, and endoscopist specialty and sex).

**Table 4:** Adjusted odds ratios for very early repeat colonoscopy among patients with polypectomy or biopsy at index procedure^#^.

		Clinical setting of index colonoscopy		
	Overall	Teaching hospital	Community hospital	Nonhospital facility
		OR (95% CI)	OR (95% CI)	OR (95% CI)
*Patient factors*				
Patient age (per one year increase)	1.02 (1.02, 1.02)	1.02 (1.01, 1.03)	1.02 (1.01, 1.02)	1.02 (1.01, 1.03)

Patient sex				
Female	0.75 (0.70, 0.79)	0.67 (0.57, 0.79)	0.71, (0.66, 0.78)	0.82 (0.74, 0.91)
Male	Reference	Reference	Reference	Reference

Patient ADG score^*∗*^	1.03 (1.02, 1.04)	1.06 (1.04, 1.09)	1.03 (1.02, 1.04)	1.02 (1.00, 1.04)

*Index colonoscopy factors*				
Setting				
Teaching hospital	1.02 (0.88, 1.19)	Not applicable	Not applicable	Not applicable
Community hospital	Reference			
Nonhospital facility	1.44 (1.30, 1.60)			

Complete	Reference	Reference	Reference	Reference
Incomplete	8.18 (7.07, 9.45)	7.76 (5.93, 10.14)	7.04 (5.75, 8.62)	11.64 (8.87, 15.27)

*Index endoscopist prior year volumes*				
Colonoscopy volume^*∗*1^	0.99 (0.98, 1.01)	0.95 (0.92, 0.97)	0.97 (0.95, 0.99)	1.02 (1.00, 1.04)

Standard polypectomy volume^*∗*1^	0.99 (0.97, 1.01)	1.06 (1.02, 1.10)	1.02 (1.00, 1.04)	0.96 (0.94, 0.98)

Volume of “removal of large polyp or obstructing lesion” ^*∗*2^	1.01 (0.98, 1.03)	1.02 (0.96, 1.08)	0.98 (0.95, 1.02)	1.02 (0.97, 1.07)

^*∗*^Per one unit increase; ^1^square root transformation; ^2^log transformation; ^#^adjusted for patient socioeconomic status, endoscopist specialty, and sex.

**Table 5:** Adjusted odds ratios for very early repeat colonoscopy with same endoscopist as at index procedure^#^.

		Clinical setting of index colonoscopy		
	Overall	Teaching hospital	Community hospital	Nonhospital facility
		OR (95% CI)	OR (95% CI)	OR (95% CI)
*Patient factors*				
Patient age (per one year increase)	0.99 (0.98, 1.00)	0.99 (0.98, 1.01)	0.99 (0.98, 1.00)	0.99 (0.98, 1.00)

Patient sex				
Female	0.93 (0.85, 1.03)	1.09 (0.85, 1.39)	0.94 (0.81, 1.09)	0.92 (0.78, 1.08)
Male	Reference	Reference	Reference	Reference

Patient ADG score^*∗*^	0.97 (0.95, 0.99)	0.96 (0.92, 1.00)	0.97 (0.94, 1.00)	0.97 (0.04, 1.00)

*Index colonoscopy factors*				
Setting				
Teaching hospital	0.80 (0.62, 1.01)	Not applicable	Not applicable	Not applicable
Community hospital	Reference			
Nonhospital facility	0.71 (0.60, 0.83)			

Complete	Reference	Reference	Reference	Reference
Incomplete	1.63 (1.37, 1.93)	1.32 (0.91, 1.91)	1.50 (1.18, 1.90)	1.22 (0.91, 1.65)

Open access	Reference	Reference	Reference	Reference
Not open access	1.10 (0.98, 1.24)	1.42 (1.03, 1.94)	0.90 (0.74, 1.10)	1.35 (1.11, 1.64)

Most complex procedure at index colonoscopy				
“Removal of large polyp or obstructing lesion”	2.33 (1.75, 3.10)	1.76 (0.83, 3.70)	3.18 (1.94, 5.21)	1.94 (1.26, 3.00)
Standard polypectomy	0.77 (0.68, 0.88)	0.91 (0.63, 1.32)	0.82 (0.69, 0.98)	0.71 (0.57, 0.87)
Biopsy	0.35 (0.29, 0.43)	0.49 (0.33, 0.74)	0.35 (0.28, 0.45)	0.30 (0.21, 0.42)
None of the above	Reference	Reference	Reference	Reference

*Index endoscopist prior year volumes*				
Colonoscopy volume^*∗*1^	1.01 (0.99, 1.03)	1.03 (0.97, 1.08)	0.99 (0.97, 1.02)	1.02 (1.00. 1.05)

Standard polypectomy volume^*∗*1^	1.04 (1.01, 1.06)	1.00 (0.93, 1.08)	1.05 (1.01, 1.08)	1.03 (0.99, 1.06)

Large or obstructing polypectomy volume^*∗*2^	1.07 (1.02, 1.11)	1.07 (0.96, 1.19)	1.09 (1.02, 1.15)	1.06 (1.00, 1.13)

^*∗*^Per one unit increase; ^1^square root transformation; ^2^log transformation; ^#^adjusted for patient socioeconomic status, endoscopist specialty, and sex.

## Data Access

The data that support the findings of this study are available from the Institute for Clinical Evaluative Sciences, the Ontario Ministry of Health and Long-Term Care, the Canadian Institute for Health Information, and Cancer Care Ontario, but restrictions apply to the availability of these data which were used under license for the current study and so are not publicly available. The data set from this study is held securely in coded form at the Institute for Clinical Evaluative Sciences (ICES). While data sharing agreements prohibit ICES from making the data set publicly available, access may be granted to those who meet prespecified criteria for confidential access, available at https://www.ices.on.ca/DAS. The full data set creation plan is available from the authors upon request.
